# Response of the Hepatic Transcriptome to Aflatoxin B_1_ in Domestic Turkey (*Meleagris gallopavo*)

**DOI:** 10.1371/journal.pone.0100930

**Published:** 2014-06-30

**Authors:** Melissa S. Monson, Robert E. Settlage, Kevin W. McMahon, Kristelle M. Mendoza, Sumit Rawal, Hani S. El-Nezami, Roger A. Coulombe, Kent M. Reed

**Affiliations:** 1 Department of Veterinary and Biomedical Sciences, College of Veterinary Medicine, University of Minnesota, Saint Paul, Minnesota, United States of America; 2 Data Analysis Core, Virginia Bioinformatics Institute, Virginia Polytechnic Institute and State University, Blacksburg, Virginia, United States of America; 3 Department of Animal, Dairy and Veterinary Sciences, College of Agriculture, Utah State University, Logan, Utah, United States of America; 4 School of Biological Sciences, University of Hong Kong, Hong Kong, China; University of California, Davis, United States of America

## Abstract

Dietary exposure to aflatoxin B_1_ (AFB_1_) is detrimental to avian health and leads to major economic losses for the poultry industry. AFB_1_ is especially hepatotoxic in domestic turkeys (*Meleagris gallopavo*), since these birds are unable to detoxify AFB_1_ by glutathione-conjugation. The impacts of AFB_1_ on the turkey hepatic transcriptome and the potential protection from pretreatment with a *Lactobacillus*-based probiotic mixture were investigated through RNA-sequencing. Animals were divided into four treatment groups and RNA was subsequently recovered from liver samples. Four pooled RNA-seq libraries were sequenced to produce over 322 M reads totaling 13.8 Gb of sequence. Approximately 170,000 predicted transcripts were *de novo* assembled, of which 803 had significant differential expression in at least one pair-wise comparison between treatment groups. Functional analysis linked many of the transcripts significantly affected by AFB_1_ exposure to cancer, apoptosis, the cell cycle or lipid regulation. Most notable were transcripts from the genes encoding E3 ubiquitin-protein ligase Mdm2, osteopontin, S-adenosylmethionine synthase isoform type-2, and lipoprotein lipase. Expression was modulated by the probiotics, but treatment did not completely mitigate the effects of AFB_1_. Genes identified through transcriptome analysis provide candidates for further study of AFB_1_ toxicity and targets for efforts to improve the health of domestic turkeys exposed to AFB_1_.

## Introduction

Consumption of feed contaminated with mycotoxins can adversely affect poultry performance and health. Mycotoxins are estimated to contaminate up to 25% of world food supplies each year [1]. Due to potent hepatotoxicity and worldwide impacts, aflatoxin B_1_ (AFB_1_) is one of the most important mycotoxins [2], [3]. The extreme toxicity of AFB_1_ in domestic turkeys (*Meleagris gallopavo*) was demonstrated in 1960, when Turkey “X” Disease caused the deaths of over 100,000 turkeys and other poultry in England as a result of feeding AFB_1_-contaminated peanut-meal [4]. High doses of AFB_1_ can cause acute mortality; exposure at lower concentrations causes loss of appetite, liver damage, and immunosuppression [3]. Chronic dietary exposure to AFB_1_ and other aflatoxins also negatively affects poultry production traits, including weight gain, feed conversion, egg production and hatchability [5], [6], [7]. Consequently, aflatoxicosis is estimated to cost the poultry industry over $143 million in losses each year [1].

The toxicity of AFB_1_ is initiated by its bioactivation into the electrophilic *exo*-AFB_1_-8,9-epoxide (AFBO) [8]. Bioactivation is mediated by cytochrome P450s (P450s) located predominantly in hepatocytes, making the liver the primary target for toxicity [8]. The high sensitivity of domestic turkeys to AFB_1_ is likely due to a combination of efficient hepatic P450s and dysfunctional alpha-class glutathione *S*-transferases (GSTAs) that cannot conjugate and detoxify AFBO [9], [10], [11]. Although the cytochrome (*CYP*) and *GSTA* genes involved in the bioprocessing of AFB_1_ have been examined in the turkey, the impact of AFB_1_ on expression of other genes is not well understood. AFBO forms adducts with DNA and RNA, which can block transcription and translation and can induce DNA mutations [2], [8], [12]. Genes directly involved in these processes are likely candidates for expression changes in response to AFB_1_, along with genes that initiate or prevent apoptosis and carcinogenesis. In liver tissue from chickens (*Gallus gallus*), AFB_1_ is known to affect genes associated with fatty acid metabolism, development, detoxification, immunity and cell proliferation [13].

Once the impact of AFB_1_ on gene expression is understood, these changes can be used to evaluate methods directed at reducing and/or preventing aflatoxicosis. Probiotic gram-positive strains of *Lactobacillus*, *Propionibacterium* and *Bifidobacterium* can bind to AFB_1_ *in vitro* [14], [15], [16]. In chickens, injection of *L. rhamnosus* strain GG (LGG), *L. rhamnosus* strain LC-705 (LC-705), and *P. freudenrieichii* strain *shermanii* JS (PJS) into the intestinal lumen has also been shown to decrease AFB_1_ absorption into duodenal tissue [15], [17]. A probiotic mixture of LGG, LC-705, and PJS has therefore been proposed as a feed additive to inhibit AFB_1_ uptake from the small intestine and attenuate AFB_1_-induced toxicity in poultry. Given their susceptibly to AFB_1_, domestic turkeys provide an ideal model to test the ability of these probiotics to reduce aflatoxicosis.

This study was designed to examine the response of the turkey hepatic transcriptome to AFB_1_ and evaluate the chemopreventive potential of *Lactobacillus*-based probiotics using high throughput RNA-sequencing (RNA-seq). Corresponding phenotypic data from this challenge trial has been characterized in another report [18]. To our knowledge, only one study on swine has used RNA-seq to investigate the impacts of AFB_1_ exposure on gene expression [19]. Therefore, this analysis provides the first detailed examination of genes involved in turkey responses to AFB_1_ and modulation of its toxicity by probiotics.

## Materials and Methods

### Ethics Statement

All *in-vivo* work, including AFB_1_ challenge trial and sample collection, was performed at Utah State University (USU) in an Association for Assessment and Accreditation of Laboratory Animal Care accredited facility according to a protocol (Number: 1001R) approved by the USU Institutional Animal Care and Use Committee. All efforts were made to minimize suffering, such as dosage that would not cause mortality and euthanasia of poults by CO_2_ asphyxiation upon completion of the study.

### Animals and Probiotic Preparation

One day-old male Nicholas domestic turkey poults (generously supplied by Moroni Feed Co., Ephraim, UT) were acclimated for 10 days at USU on a corn-based commercial diet (Moroni Feed Co.). The challenge trial was performed on young poults, rather than adults, since the activity of P450s and AFBO production is inversely related to age [20]. A probiotic mixture of lyophilized bacteria from Valio Ltd. (Helsinki, Finland) was used in the challenge trial. This mixture contained 2.3×10^10^ CFU/g of *L. rhamnosus* GG, 3.0×10^10^ CFU/g of *L. rhamnosus* LC-705, 3.5×10^10^ CFU/g of *Propionibacterium freunchdenreichii* sp. shermani JS, and 2.9×10^10^ CFU/g of *Bifidobacterium* sp., along with 58% microcrystalline cellulose, 27% gelatin and magnesium salt. Probiotic (PB) solution was prepared by directly suspending bacteria in phosphate buffered saline at a final concentration of 1×10^11^ CFU/mL as previously described [21].

### AFB_1_ Challenge Trial

Poults (N = 40) were randomly assigned to one of 4 treatment groups (n = 10/group) (phosphate buffered saline control (CNTL), probiotic mixture (PB), aflatoxin B_1_ (AFB), and probiotic + aflatoxin B_1_ (PBAFB). After the 10 day acclimation period, turkeys in the PB and PBAFB groups were given 0.5 mL of PB (5×10^10^ CFU) daily by oral gavage from day 11 to day 31. Birds in the CNTL and AFB groups were administered 0.5 mL of phosphate buffered saline by oral gavage on day 11–31. The corn-based starter diet was fed to all poults in all treatments from day 11 to day 20. On day 21–31, turkeys in the CNTL and PB groups continued to receive the unaltered feed, while 1 ppm AFB_1_ was introduced into the diet fed to birds in the AFB and PBAFB groups. Poults were euthanized by CO_2_ asphyxiation on day 31. Liver samples were collected directly into RNAlater (Ambion, Inc., Austin, TX), perfused overnight at 4°C, and then stored at −20°C to preserve RNA. Phenotypic effects of aflatoxicosis, including weight gain, liver weight, histopathology, and serum analysis for this challenge trial are presented elsewhere [18]. Aflatoxicosis was verified by these measures for individuals in the AFB_1_-treated groups.

### RNA Isolation and Sequencing

Total RNA was isolated by TRIzol extraction (Ambion, Inc.) from 3 tissue samples/treatment group (n = 12) and stored at −80°C to prevent degradation. gDNA contamination was removed from each RNA sample with the Turbo DNA-*free*™ Kit (Ambion, Inc.). RNA concentration and quality were assessed by denaturing gel electrophoresis and Nanodrop 1000 spectrophotometer (Nanodrop Technologies, Wilmington, DE). For each treatment group, individual DNase-treated RNA samples were pooled (n = 3) in equimolar amounts and RNA concentration in each pool was verified by spectrophotometry. Samples were pooled to maximize the depth of sequence collected from each treatment group, including rare sequences. Total RNA samples from the CNTL and AFB groups (8.5 µg) and the PB and PBAFB groups (6 µg) were submitted for sequencing on the Illumina Genome Analyzer II at the Mayo Clinic (Rochester, MN). Four libraries (1 library/treatment group) were constructed according to the Illumina mRNA Sequencing Protocol. RNA integrity for each library was confirmed with the 2100 Bioanalyzer (Agilent Technologies, Santa Clara, CA). Libraries were run on 4 flow cell lanes to produce 51 bp single-end reads. Sequencing at this depth required 2 flow cells (CNTL and AFB groups on flow cell 1 and PB and PBAFB on flow cell 2).

### Read Filtering, Trimming, and Dataset QC Analysis

RNA-seq datasets for each library were filtered by BLAST aligning reads against common contaminating sequences, including bacteria gDNA and Illumina sequencing adaptors/primers. Using CLC Genomics Workbench (CLC bio, Cambridge, MA), reads were then trimmed for low quality (limit 0.05 for error probability, maximum of 2 ambiguities) and end trimmed (4 terminal bases on both 5′ and 3′ ends) to reduce library base composition biases and end quality dips. FastQC [22] was utilized to examine dataset quality before and after the trimming and filtering protocols.

### *De novo* Assembly

The Velvet [23] and Oases [24] pipelines were used for *de novo* assembly of the corrected reads from all four datasets into predicted transcripts. Multiple sub-assemblies were generated in Velvet and Oases using a range of k-mer (hash) lengths (21, 23, 25, 27, 29, and 31) to construct contigs. A final merged assembly was created using the contigs from all six sub-assemblies as input sequence for Velvet and Oases with a k-mer value of 27. Default parameters were utilized for all assemblies, with the cutoffs for contig coverage and connection support set at 3. Corrected reads were mapped back to the final assembled predicted transcripts using BWA [25]. Counts of reads uniquely mapping to each transcript were determined using HTSeq in intersect-nonempty mode [26]. Reads that mapped to multiple transcripts were not included in coverage counts.

### Transcript Annotation

Predicted transcripts were annotated by three BLAST alignments. Transcripts were first compared to cDNAs from the turkey genome build UMD 2.01 (www.ensembl.org) and assigned their coordinating NCBI Transcript Reference Sequence (RefSeq) IDs. A similar search of the chicken genome (Galgal 4.0) identified matches to chicken RefSeq mRNAs and a final BLAST comparison was performed to the UniProtKB Swiss-Prot protein database. For all three searches, BLAST hits were considered valid for bit scores ≥100 and the top hits were recorded. Transcripts that showed significant differential expression (DE) but lacked hits from the transcriptome-wide BLAST search were then aligned to the NCBI non-redundant nucleotide (NR) database. This allowed identification of un-annotated but previously characterized cDNAs, non-protein coding RNAs and other sequences only accessioned in the NR database.

### Transcript Coverage Filtering

A coverage threshold of 0.1 read/million mapped was applied to filter predicted transcripts for sufficient read depth. To account for differences in the total number of mapped reads per treatment group, the minimum number of reads that must map to each transcript was determined separately in each treatment (Table S1). Transcripts were included in the transcriptome content and numbers for any treatment group in which they met this coverage threshold; lowly expressed transcripts were excluded only from the transcript list in the treatment(s) in which they fell below this threshold.

### Differential Expression Analysis

Expression of each transcript in each treatment group was determined from read counts normalized with size scaling using the R package DESeq [27]. Since datasets were derived from RNA pools, DESeq estimated the within-treatment variation in expression of each transcript using its mean and the dispersion of its expression across all treatment groups (method  =  “blind” and sharingMode  =  “fit-only” settings). To prevent skewing of the means and variance estimates, all predicted transcripts were analyzed by DESeq rather than just the filtered set. Pair-wise comparisons for statistical significance based on a negative binomial distribution were made in DESeq using the mean and dispersion estimates and p-values were assigned. Expression in each treatment was compared to the CNTL group to determine the impact of AFB_1_ and/or PB. Two additional contrasts of the PBAFB group with the AFB and PB groups were also performed to investigate the ability of PB to mitigate AFB_1_ effects. Transcripts were considered to have significant DE if q-values (FDR adjusted p-values based on the Benjamin-Hochberg procedure) were ≤0.05. Scatter plots and heat maps generated in R were used to visualize the datasets and results of the expression analyses. Venn diagrams were created using a combination of BioVenn [28], Venny [29] and the R package *VennDiagram* 1.6.4 [30].

### Genome and Functional Analysis

Filtered transcripts were aligned to the domestic turkey genome build UMD 2.01 using GMAP [31]. Gene Ontology (GO) terms associated with significant DE transcripts were determined using Blast2GO V.2.6.6 [32], [33]. Further functional characterization of these DE transcripts was performed using Ingenuity Pathway Analysis (IPA) (Ingenuity Systems, Redwood City, CA).

## Results

### RNA-seq Datasets

Sequencing of the four pooled libraries produced over 356 M 51 bp reads with an average quality score of 32.4 (Table 1) (as part of SRA project ID: SRP042724). Libraries run on the same flow cell generated similar read numbers, with 75 M reads collected for the CNTL library (SRX566381), 65 M for AFB (SRX569978), 111 M for PB (SRX570327) and 105 M for PBAFB (SRX570328). The number of sequence reads varied between flow cells, with more than 76 M additional reads produced on the second flow cell. Given this variation, normalization for library size was critical for accurate expression analyses. After read trimming and filtering (33.9 M reads removed), the corrected datasets were reduced by an average of 8.5 M reads. Average read length decreased to 42.9 bp, while average quality score per read increased to 33.3. Quality scores remained lower for reads collected on the first flow cell (CNTL and AFB datasets) than the second (PB and PBAFB) even after filtering and trimming (Figure S1). Box-plots demonstrate that the quality scores across base position in each corrected dataset were sufficiently high for reliable base calling (Figure S2). Cumulatively, all corrected reads comprise 13.8 Gb of usable sequence for transcriptome assembly (Table 1).

**Table 1:** Descriptive statistics for liver RNA-seq datasets.

			Number of Reads^1^					
Filtering and Trimming	Average Read Length	Average Quality Score	CNTL	AFB	PB	PBAFB	Total	Total Sequence
Before	51 bp	32.4	75,218,798	64,798,923	111,295,859	105,158,981	356,472,561	18.2 Gb
After	42.9 bp	33.3	66,213,757	55,553,255	103,367,767	97,459,919	322,594,698	13.8 Gb
Discarded	51 bp	ND^2^	9,005,041	9,245,668	7,928,092	7,699,062	33,877,863	4.4 Gb

1 Treatment groups are control (CNTL), aflatoxin B_1_ (AFB), probiotic mixture (PB), and probiotic + aflatoxin B_1_ (PBAFB).

2 Not determined (ND).

### *De novo* Transcriptome Assembly

Final assembly of the transcriptome via the Velvet and Oases pipelines utilized 95.2% of the groomed RNA reads and generated 211 Mb of potential expressed sequence (Table 2). The assembly contains 174,010 predicted transcripts ranging in size from 200 to 39,213 bp. This number decreased to 169,387 transcripts after filtering out transcripts with insufficient coverage (Table 2; Table S1). Interestingly, the coverage threshold of 0.1 read/million mapped coincided with the most frequent read depth in each treatment (Figure S3). Although this filtering kept 99.9% of mapped reads, between 6.8% and 13% of expressed predicted transcripts fell below the threshold in each treatment group (Table S1).

**Table 2:** Summary of the *de novo* liver transcriptome assembly.

		Total Assembled	Above Coverage Threshold^1^
Number of Reads	Mapped	307,105,226 (95.2%)	306,815,840 (95.1%)
	Unmapped	15,489,472 (4.8%)	15,778,858 (4.9%)
Total Number of Predicted Transcripts		174,010	169,387
Transcript Length (bp)	Min	200	200
	Mean	1,213	1,238
	Max	39,213	39,213
	N50	2,038	2,052
Total Residues (bp)		211,012,448	209,738,998
Average GC Content/Transcript		46.9%	46.9%
Transcripts Identified	Turkey	85,435 (49.1%)	85,052 (50.2%)
	Chicken	102,421 (58.9%)	101,831 (60.1%)
	Swiss-Prot	78,167 (44.9%)	78,009 (46.1%)
	Total Known	108,161 (62.2%)	107,503 (63.5%)
	Unknown	65,849 (37.8%)	61,884 (36.5%)

1 Predicted transcripts were filtered according to a coverage threshold of 0.1 read/million mapped.

Fitting expectations for the turkey transcriptome, transcripts that met the coverage threshold had an N50 of 2.1 Kb and a GC content of 46.9% (Table 2). Mean filtered transcript length was 1.2 Kb, which is shorter but consistent with the average size of cDNAs in the turkey (1.7 Kb), chicken (2.5 Kb), duck (1.7 Kb) and zebra finch (1.4 Kb) Ensemble gene sets (genome assemblies UMD 2.01, Galgal 4.0, BGI duck 1.0 and taeGut 3.2.4). The majority (87.0%) of filtered liver transcripts ranged from 250 bp to 4 Kb (Figure S4). The few overly large transcripts have BLAST hits to known genes, but also contain repetitive sequences and expressed retrotransposons like CR1 repeats and LTR-elements. These large constructs were generated because the repeat-containing reads from across the genome cannot be uniquely distinguished during assembly even if discarded in mapping.

**Figure 1:**
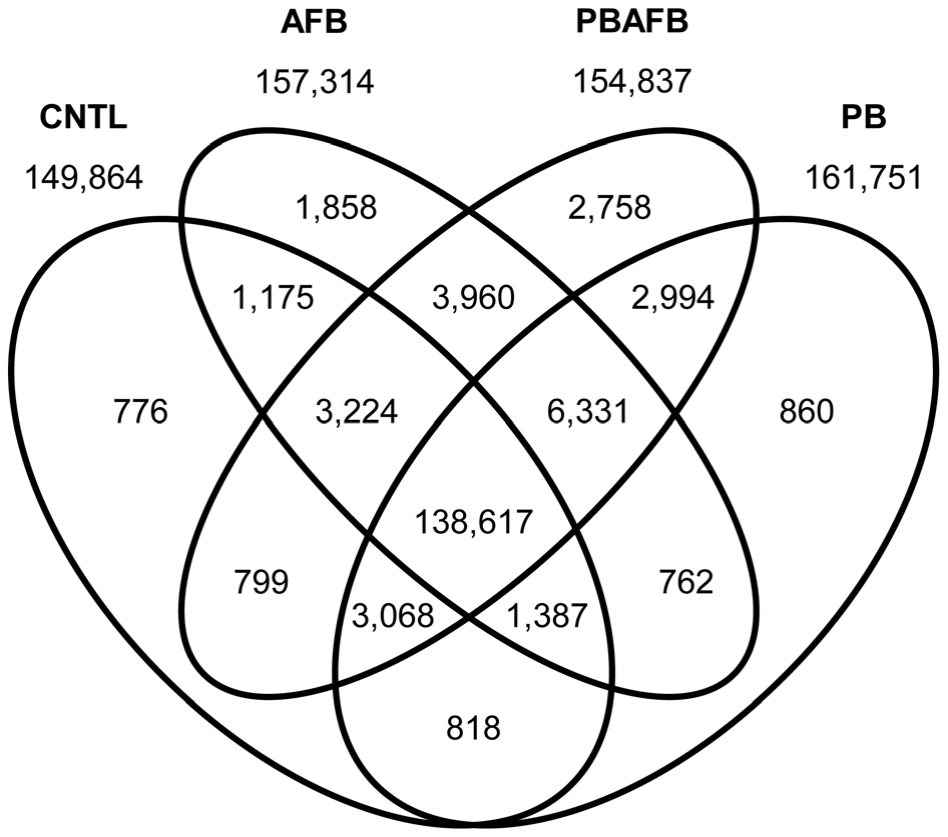
Comparative transcriptome content in domestic turkey liver. The number of transcripts shared or unique to each combination of treatments after filtering is indicated in each section of the diagram. Totals for the control (CNTL), aflatoxin B_1_ (AFB), probiotic mixture (PB), and probiotic + aflatoxin B_1_ (PBAFB) groups are shown above each ellipse.

Most filtered transcripts (81.8%) were represented in all datasets; however, 24,518 (14.5%) were shared between only two or three treatments and 6,252 (3.7%) were unique to a single treatment (Figure 1). Of these unique transcripts, 76.9% did not match to previously annotated genes. BLAST screening identified only 63.5% of all filtered transcripts (Table 2). Although only 50.2% of filtered transcripts matched to known turkey mRNAs, 89.4% of transcripts mapped to the turkey genome (Table S2). This difference suggests that the majority of unknown transcripts represent splice variants, unannotated genes, and non-protein coding RNAs. Therefore, mapped transcripts provide a resource for genome annotation and improvement of gene models. The number of mapped transcripts per Mb of chromosome can be used to predict gene density. Microchromosomes, although small, were especially gene-rich. MGA18 and MGA27 had inflated relative gene content due to their poor representation in the genome assembly.

### Differential Expression and Functional Analysis

Pair-wise comparisons of expression for predicted transcripts were performed using DESeq to normalize read counts, estimate dispersions, and perform significance tests. Since individuals were pooled prior to library construction, DESeq estimated within-group expression variance for each transcript using the relationship between the mean and the dispersion across all conditions. This decreases power and may limit to some extent the ability to identify significant differences in transcript abundance; estimation also increases type 1 errors. Therefore, the 803 transcripts with significant DE in at least one between-group comparison likely represent a subset of the total influenced by each treatment. Read counts from HTSeq, results from DESeq, BLAST annotations, and associated GO terms are provided for significant DE transcripts in each pair-wise comparison in Table S3. Data from DE analysis and BLAST screening for the complete list of predicted transcripts is available upon request.

**Figure 2:**
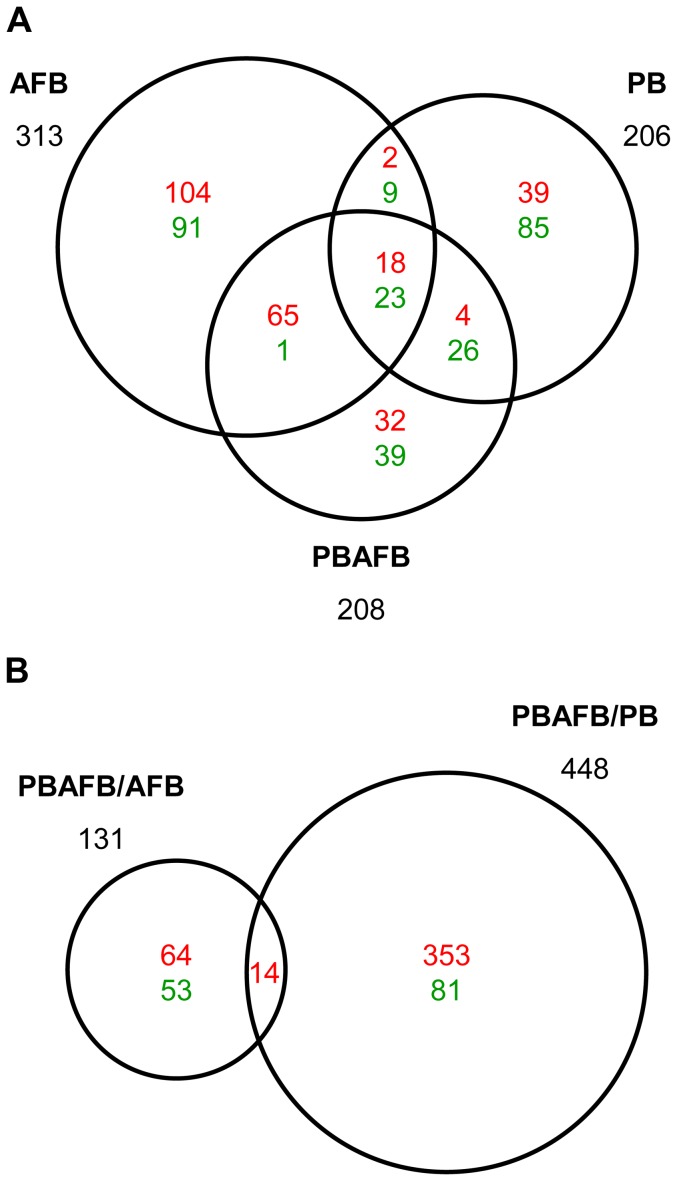
Liver transcripts with significant DE in each comparison between treatment groups. Numbers in each section indicate predicted transcripts with significant differential expression (DE) (q-value ≤0.05) that are shared between or unique to each comparison. Up-regulated transcripts are shown in red and down-regulated are shown in green. The total number of significant transcripts for each comparison is shown beside the corresponding circle. (A) Transcripts with significant DE when compared to the control (CNTL) group. (B) Transcripts with significant DE in inter-treatment comparisons (i.e. the probiotic + aflatoxin B_1_ (PBAFB) group compared to the aflatoxin B_1_ (AFB) or probiotic mixture (PB) group).

In pair-wise comparisons to the CNTL group, 538 transcripts were identified with significant DE in at least one other treatment (Figure 2A). Only 41 transcripts had significant DE in all three treatments (AFB, PB and PBAFB), including transcripts from apolipoprotein A-IV (*APOA4*) and alpha-2-macroglobulin (*A2M*). BLAST annotated 77.9% of these significant DE transcripts, including matches to sequences in the NR database (Table S3). Despite relatively high BLAST identification, Biological Process GO-terms could only be associated with 37.7% of these significant DE transcripts.

**Figure 3:**
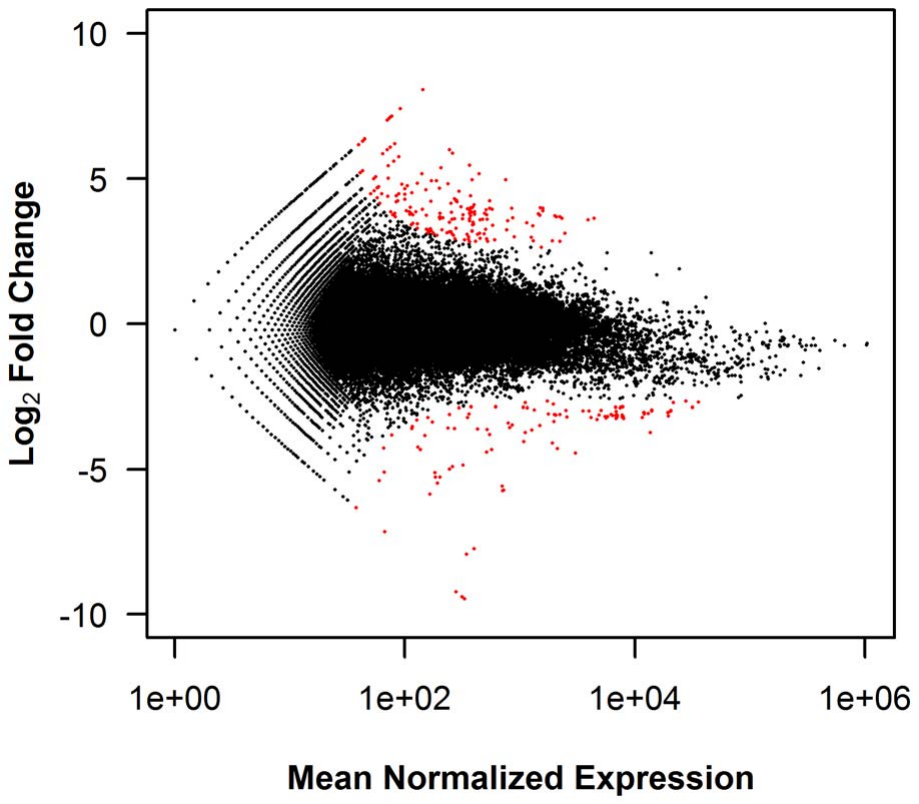
Relationship between mean expression and log_2_ FC in the AFB to CNTL comparison. Log_2_ fold change (FC) was plotted against the mean normalized read counts for each predicted transcript with non-zero expression values in both the control (CNTL) and aflatoxin B_1_ (AFB) treatments. As determined in DESeq [27], transcripts with significant differential expression (DE) (q-values ≤0.05) are highlighted in red.

To visualize the significant transcripts, log_2_ fold change was plotted against mean normalized expression for each predicted transcript (Figure 3; Figure S5). In the AFB to CNTL comparison, transcripts with significant DE are located nearer the asymptotic curves for fold change in both the positive and negative directions (Figure 3). As mean normalized expression values decrease, expression changes must increase for transcripts to be significant. The same relationship for significance is demonstrated in all comparisons between treatment groups (Figure S5).

**Figure 4:**
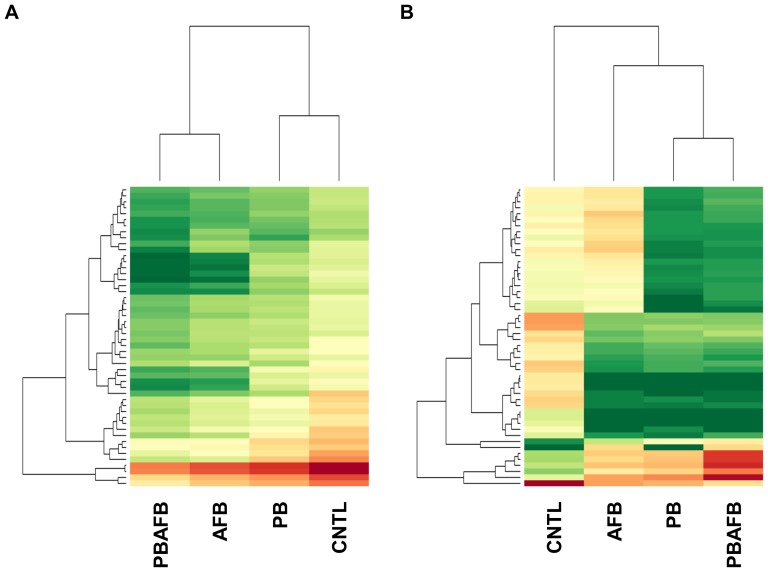
Comparative expression of select transcripts across four treatment groups. Heat maps were generated from variance stabilized and normalized read counts using DESeq [27] across the control (CNTL), aflatoxin B_1_ (AFB), probiotic mixture (PB), and probiotic + aflatoxin B_1_ (PBAFB) groups. Expression level for each transcript is represented by a color range from green (low expression) to red (high expression). (A) 50 transcripts with the highest expression across all treatments. (B) 50 transcripts with the most highly significant differential expression (DE) in pair-wise comparisons to the CNTL.

Relative similarity between the four treatments depends on the transcripts selected for comparison. When the 50 transcripts with highest expression levels are compared, the treatments divide into two clusters (CNTL and PB, and AFB and PBAFB) with highest expression values in the CNTL (Figure 4A). When comparing expression of the 50 transcripts with the greatest significant DE, the PB and PBAFB groups cluster (Figure 4B). Expression in the AFB group shares similarities with both this cluster and the CNTL group. Beyond these overall trends, each pair-wise comparison also illustrates specific effects of AFB_1_ and probiotic treatments on expression.

### AFB versus CNTL

**Figure 5:**
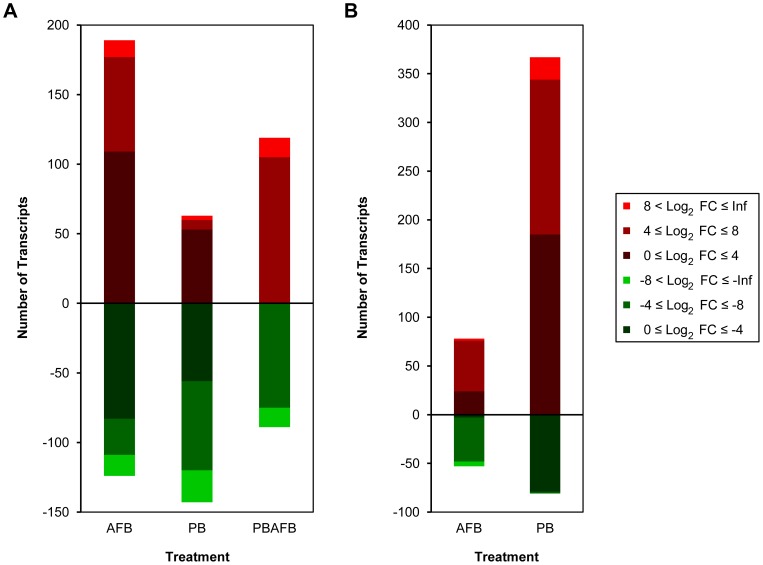
Magnitude of expression changes in transcripts with significant DE in each comparison between treatments. The number of significantly up- or down-regulated transcripts in each range of log_2_ fold change (FC) is illustrated for each pair-wise comparison. (A) Transcripts with significant differential expression (DE) in the aflatoxin B_1_ (AFB), probiotic mixture (PB) and probiotic + aflatoxin B_1_ (PBAFB) groups compared to the control (CNTL) group. (B) Transcripts with significant DE in PBAFB compared to AFB and PB.

Comparison of expression in the AFB and CNTL groups identified 144,403 shared transcripts after threshold filtering (Figure 1). Expression of 313 predicted transcripts was significantly affected by AFB_1_ treatment (Figure 2A); this is a greater number of significant DE transcripts than observed in any other treatment group (PB or PBAFB, see below). In the AFB group, 60.4% of significant DE transcripts were up-regulated (Figure 2A; Figure 5A), with large log_2_ fold changes seen in transcripts from keratin 20 (*KRT20*), cell-death activator CIDE-3 (*CIDEC*), and E3 ubiquitin-protein ligase Mdm2 (*MDM2*). DE was most significant for transcripts from S-adenosylmethionine synthase isoform type-2 (*MAT2A*) and *A2M* (q-value  = 3.22E-11). BLAST identified genes corresponding to 50.5% of transcripts with significant DE in the AFB group (Table S3). When expanded to include all sequences in the NR database, annotation increased to 88.8%, primarily due to hits to uncharacterized cDNAs.

**Figure 6:**
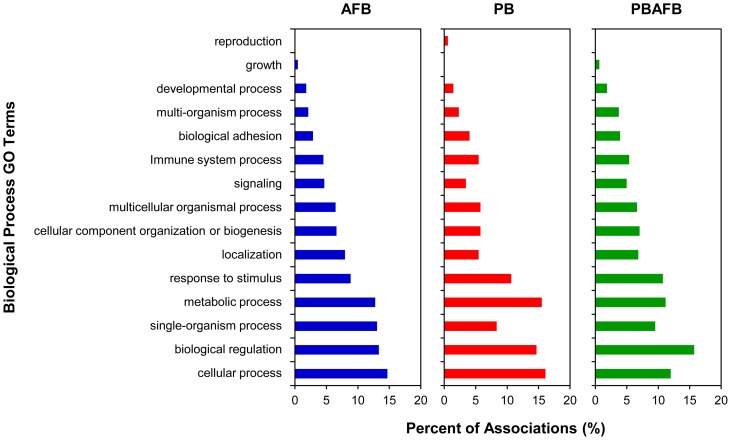
Biological process GO terms associated with significant DE transcripts in treatments compared to CNTL. For each pair-wise comparison to the control (CNTL) group, level 2 biological process Gene Ontology (GO) terms were matched to transcripts with significant differential expression (DE) using BLAST2GO [32]. The distribution of associated GO terms for significant transcripts in the aflatoxin B_1_ (AFB), probiotic mixture (PB), and probiotic + aflatoxin B_1_ (PBAFB) groups was plotted as the percent of total associations.

Associations to level 2 Biological Process GO terms were made for 132 significant DE transcripts (42.2%) (Table S3). Cellular process, single-organism process and biological regulation were the most often associated GO terms (Figure 6). As a parent term for apoptotic GO terms, “single-organism process” occurred more frequently in the AFB group (13.0%) than in any other treatment. Many (21.4%) significant DE transcripts have homology to genes with known function in carcinogenesis or apoptosis. The most significant of these transcripts are shown in Table 3. Other transcripts with significant DE in the AFB group had homology with genes involved in lipid metabolism or accumulation. For example, lipoprotein lipase (*LPL*) and MID1 interacting protein 1 (*MID1IP1*) were significantly down-regulated only in the AFB group (Table 4).

**Table 3:** Transcripts involved in carcinogenesis and/or apoptosis with significant DE in treatment comparisons to CNTL.

			AFB		PB		PBAFB	
Transcript ID	BLAST Hit Name^1^	Symbol	Log_2_ FC	q-value	Log_2_ FC	q-value	Log_2_ FC	q-value
Locus_921_Transcript_5	alpha-2-macroglobulin^2^	*A2M*	−9.40	3.22E–11	−10.10	1.05E–21	−8.34	2.45E–06
Locus_921_Transcript_6	alpha-2-macroglobulin^2^	*A2M*	−9.47	3.22E–11	−8.17	5.07E–20	−10.41	2.56E–07
Locus_5_Transcript_11597	aldolase B, fructose-bisphosphate^2^	*ALDOB*	−2.66	0.0418	−3.04	1.28E–05	−5.10	0.00266
Locus_1135_Transcript_8	aldolase B, fructose-bisphosphate^2^	*ALDOB*	−3.15	0.00365	−0.14	1.00	−2.18	1.00
Locus_66538_Transcript_2	BAG family molecular chaperone regulator 4	*BAG4*	3.11	1.00	3.54	0.322	6.99	9.02E–05
Locus_66856_Transcript_6	cell-death activator CIDE-3^2^	*CIDEC*	3.81	0.0027	2.91	0.0233	5.21	0.00327
Locus_212_Transcript_13	CDK inhibitor CIP1^2^	*CIP1*	5.47	7.85E–07	1.04	1.00	6.23	1.78E–04
Locus_158_Transcript_290	ceruloplasmin^2^	*CP*	3.12	0.0418	−0.30	1.00	1.99	1.00
Locus_7438_Transcript_1	cathepsin E	*CTSE*	−3.11	0.0238	−0.91	1.00	−4.90	0.0133
Locus_11901_Transcript_17	deiodinase, iodothyronine, type II^2^	*DIO2*	−1.62	1.00	−1.43	1.00	−4.84	0.0106
Locus_55440_Transcript_3	deleted in malignant brain tumors 1^2^	*DMBT1*	INF	0.417	INF	1.00	INF	0.00773
Locus_53943_Transcript_1	S-adenosylmethionine synthase isoform type-2	*MAT2A*	INF	3.22E–11	NE	N/A	INF	1.69E–06
Locus_196_Transcript_166	E3 ubiquitin-protein ligase Mdm2^2^	*MDM2*	4.24	7.68E–05	0.38	1.00	4.49	0.0161
Locus_3434_Transcript_9	E3 ubiquitin-protein ligase Mdm2^2^	*MDM2*	6.30	0.0114	1.41	1.00	7.67	0.00188
Locus_3773_Transcript_3	osteopontin^2^	*OPN*	3.61	0.00353	1.14	1.00	3.30	0.516
Locus_121_Transcript_17	phosphoinositide-3-kinase, catalytic subunit alpha^2^	*PIK3CA*	−3.81	4.49E–04	1.70	0.233	0.72	1.00
Locus_12205_Transcript_1	TAK1-like protein	*TAK1L*	4.81	0.00187	0.31	1.00	3.75	1.00

Putative functions were identified for transcripts with significant differential expression (DE) (q-value ≤0.05) using IPA, BLAST2GO or through primary literature. Log_2_ fold change (FC) and q-values were determined for transcripts in the aflatoxin B_1_ (AFB), probiotic mixture (PB), and probiotic + aflatoxin B_1_ (PBAFB) groups compared to the control (CNTL) group using DESeq [27]. Non-significant transcripts (q-value >0.05) are shown in grey.

1 See Table S3 for complete BLAST annotation for these transcripts.

2 Multiple transcripts had significant DE for these genes; only most significant for each pair-wise comparison is shown.

**Table 4:** Transcripts involved in steatosis or lipid metabolism with significant DE in treatment comparisons to CNTL.

			AFB		PB		PBAFB	
Transcript ID	BLAST Hit Name^1^	Symbol	Log_2_ FC	q-value	Log_2_ FC	q-value	Log_2_ FC	q-value
Locus_108_Transcript_6	apolipoprotein A-IV^2^	*APOA4*	3.98	9.93E–05	2.80	2.44E–04	5.40	7.63E–04
Locus_108_Transcript_13	apolipoprotein A-IV^2^	*APOA4*	3.63	0.0023	4.20	6.39E–09	6.72	1.40E–05
Locus_5_Transcript_12840	glucose-6-phosphatase, catalytic subunit^2^	G6PC*	3.74	2.99E–04	0.20	1.00	1.00	1.00
Locus_5083_Transcript_14	lipoprotein lipase^2^	*LPL*	−2.85	0.0409	0.21	1.00	−2.00	1.00
Locus_17578_Transcript_1	MID1 interacting protein 1	*MID1IP1*	−4.04	8.95E–05	1.53	1.00	2.56	1.00
Locus_54740_Transcript_1	phosphoenolpyruvate carboxykinase 1	*PCK1*	3.64	0.00478	4.39	7.86E–09	6.12	1.31E–04

Putative functions were identified for transcripts with significant differential expression (DE) (q-value ≤0.05) using IPA, BLAST2GO or primary literature. Log_2_ fold change (FC) and q-values were determined for transcripts in the aflatoxin B_1_ (AFB), probiotic mixture (PB), and probiotic + aflatoxin B_1_ (PBAFB) groups compared to the control (CNTL) group using DESeq [27]. Non-significant transcripts (q-value >0.05) are shown in grey.

1 See Table S3 for complete BLAST annotation for these transcripts.

2 Multiple transcripts had significant DE for these genes; only most significant for each pair-wise comparison is shown.

Although the liver is the site of AFB_1_ bioprocessing, none of the transcripts associated with *CYP* and *GSTA* genes had significant DE in the AFB group. Almost no change in expression was observed for transcripts from *CYP1A5* and *CYP3A37*, which encode the two liver P450s that activate AFB_1_ [11] (average log_2_ fold changes of −0.01 and −0.16). Expression changes were also minimal for five of the six *GSTA* genes (averages of 0.16, 0.25, 0.24, 1.38, and 0.39 for *GSTA1.1*, *GSTA1.2*, *GSTA2*, *GSTA3*, and *GSTA4*). No transcripts matched to the *GSTA1.3* gene in any treatment group.

### PB versus CNTL

The PB and CNTL groups share the fewest number of filtered transcripts (143,890) (Figure 1), yet only 206 significant DE transcripts were identified in the PB treatment group (Figure 2A). BLAST annotated 46.6% (58.3% with NR sequences) of these transcripts (Table S3). Unlike the AFB group, significant DE transcripts in the PB group are predominantly down-regulated (Figure 2A; Figure 5A), leading to a significantly lower mean log_2_ fold change than in the AFB_1_-treated groups (AFB or PBAFB, Figure S6A). Some down-regulated transcripts, including many from *A2M*, had higher significance (smaller q-values) than any transcripts in the AFB_1_-treated groups (Figure S7A, B, C).

Associations were made between 41.3% of these significant DE transcripts and Biological Process GO terms (Table S3). The most frequently associated level 2 terms were cellular process, metabolic process, and biological regulation (Figure 6). Transcripts from *CYP2H1* and poly(U)-specific endoribonuclease-A-like (*ENDOU*) were significantly down-regulated in the PB group (Table S3), illustrating the impact of probiotics on metabolic and enzymatic functions. Unlike the AFB_1_-treated groups (AFB and PBAFB), only 9.7% of significant transcripts in PB had links to cancer and these were almost exclusively transcripts from *A2M*. Since these transcripts were significantly down-regulated in all three treatment groups (Table 3), *A2M* is unlikely to be involved in AFB_1_ toxicity.

### PBAFB versus CNTL

A total of 145,708 filtered transcripts were shared between the PBAFB and CNTL groups (Figure 1), exceeding those found for both the AFB and PB groups. Only 208 predicted transcripts had significant DE in the PBAFB group (Figure 2A) and BLAST successfully identified 66.8% (80.3% with NR sequences) of these transcripts (Table S3). More than half (51.4%) were shared with the AFB group, but only 34.1% were significantly DE in both PBAFB and PB (Figure 2A). Similar to the AFB group, the majority (57.2%) of transcripts with significant DE were up-regulated (Figure 2A; Figure 5A). Larger changes in expression were required for significant DE in the PBAFB group, than in either AFB or PB (Figure 5A). Transcripts with significant DE in the PBAFB group more closely resembled those of the AFB group, indicating an AFB_1_ treatment effect.

Functional analysis found GO term associations for 42.3% of significant DE transcripts (Table S3) and identified further similarities between PBAFB and the other treatment groups. As in the PB group, biological regulation, metabolic process, and cellular process were the most commonly associated level 2 GO terms in the PBAFB group (Figure 6). Localization, single-organism process, and to a lesser extent signaling and cellular component organization or biogenesis GO terms occurred at higher proportions in the AFB and PBAFB groups. Increased GO associations with the “multi-organism process” term were observed in only the PBAFB group. Including transcripts from *MAT2A* and *MDM2*, 27.8% of significant DE transcripts in PBAFB were annotated to genes with known roles in cancer or apoptosis. Greater up-regulation was observed in the PBAFB group than in the AFB group for many significant transcripts involved in carcinogenesis or lipid regulation (*CIDEC*, CDK inhibitor CIP1 (*CIP1*), *MDM2*, Table 3; *APOA4*, phosphoenolpyruvate carboxykinase 1 (*PCK1*), Table 4).

### Inter-treatment Comparisons

Additional inter-treatment comparisons (PBAFB vs. AFB and PBAFB vs. PB) identified 565 transcripts with significant DE (Table S3). Most (353) of the 448 transcripts with significant DE in PBAFB vs. PB were up-regulated (Figure 2B; Figure 5B). Highly up-regulated transcripts from *MAT2A*, *CIP1*, and *MDM2* also had some of the highest significance values. Nearly 60% of significant DE transcripts in the PBAFB group compared to the AFB group were also up-regulated, but the transcripts with the highest significance were down-regulated and could not be BLAST annotated (Figure S7D). Comparison of the AFB_1_-treated groups (PBAFB vs. AFB) also found larger decreases in expression than observed in PBAFB vs. PB (Figure 5B, Figure S6B). Gene expression in the PBAFB group more closely resembled the AFB group, with 152,132 shared transcripts. A smaller number of transcripts (151,010) were shared between the PBAFB and PB groups. Together these suggest that the combined treatment (PBAFB) is more similar to treatment with AFB_1_ than probiotics.

Exposure to AFB_1_ (AFB and PBAFB groups) initiated expression of cancer-associated transcripts (such as *MAT2A*) not observed in the CNTL or PB groups. Fourteen transcripts, including expressed sequences from collagen, type II, alpha 1 (*COL2A1*) and BAG family molecular chaperone regulator 4 (*BAG4*), were significantly up-regulated in PBAFB in comparisons to both AFB and to PB indicating a synergistic effect. “Metabolic process” was most commonly associated GO term for significant DE transcripts in the PBAFB verses AFB comparison (Figure S8). For example, a transcript from *CYP51A1* was significantly up-regulated in the PBAFB group compared to the AFB group (Table S3). A higher proportion of associations to the GO terms developmental process, multicellular organismal process, and response to stimulus was identified when comparing AFB_1_-treated groups (PBAFB vs. AFB) than probiotic-treated (PBAFB vs. PB). When comparing the probiotic treated groups, the GO terms biological regulation, cellular component organization or biogenesis, localization, and signaling occurred more frequently, illustrating the impact of AFB_1_ exposure on apoptotic, cell cycle and other regulatory genes. Further investigation of the hepatic functions and pathways of these significant transcripts in the domestic turkey will be necessary to determine molecular mechanism by which AFB_1_ initiates carcinogenesis and lipid misregulation in poultry.

## Discussion

Along with providing a first characterization of the turkey liver transcriptome, this study identified several genes potentially affected by exposure to AFB_1_ and probiotics. Despite their known role in AFBO production [11], expression of *CYP1A5* and *CYP3A37* did not change in the AFB_1_-treated groups. However, probiotics influenced expression of transcripts from other *CYP* gene family members. AFB_1_ exposure also had no significant impact on expression of *GSTA* genes. Domestic turkey hepatic GSTAs are unable to conjugate AFBO *in vivo*, yet these enzymes have activity when heterologously expressed in *E. coli* [9], [10]. Hence, gene silencing mechanisms or post-transcriptional modifications are likely responsible for this dysfunction [9], either of which is consistent with the absence of significant DE in *GSTA* transcripts. Invariable expression in these *CYP* and *GSTA* genes means that the transcriptional response to AFB_1_ is mediated through genes not previously linked to aflatoxicosis in the domestic turkey.

Changes in transcript abundance were quantified through RNA-seq and novel genes in the liver transcriptome were associated with exposure to AFB_1_ and/or probiotics. Functional analysis of the significantly affected transcripts identified three major impacts: effects of AFB_1_ on genes linked to cancer, effects of AFB_1_ on genes involved in lipid metabolism, and opposing effects of PB and the combined PBAFB treatment.

### AFB_1_ and Cancer

The carcinogenic nature of AFB_1_ in mammals is well established and chronic exposure is an established risk factor for hepatocellular carcinoma in humans [2], [8]. In poultry, both acute and chronic AFB_1_ consumption cause lesions in the liver, including necrotic hepatocellular loci, focal hemorrhages, and fatty vacuolation of hepatocytes [5], [6], [34], [35]. Dietary AFB_1_ is also mutagenic in chickens, turkeys and other poultry, first generating biliary hyperplasia, followed by fibrosis and nodular tissue regeneration during long-term exposure [5], [6], [34], [35]. Histological analysis on liver sections from the AFB group identified, on average, 5–30% necrotic hepatocytes and moderate biliary hyperplasia [18]. Adverse effects on the liver from AFB_1_ exposure are likely driven by genes associated with the cycle cell and apoptosis.

Differential expression analysis of the turkey liver transcriptome identified a large number of transcripts derived from genes with known links to liver cancer in mammals. The strongest down-regulation was observed in transcripts from *A2M*, which encodes a proteinase inhibitor. *A2M* has been associated with human and rat hepatocellular carcinoma (HCC), but reports conflict on whether its expression is up- or down-regulated [36], [37], [38]. AFB_1_ has also been shown to decrease A2M secretion from rat hepatocytes [39]. However, it is unlikely that A2M is a driver of AFB_1_ toxicity in the turkey, since *A2M* transcripts were down-regulated in all three treatments, including the PB group.

Most transcripts with significant DE in the AFB group were up-regulated. Large increases in expression were observed for *MDM2*, which encodes an E3 ubiquitin-protein ligase that acts on p53, causing its degradation by the proteosome. In human lung cells expressing P450s, AFB_1_ has been shown to cause a concentration-dependent increase in *MDM2* expression [40]. Overexpression and polymorphisms in *MDM2* have been linked to human HCC [41], [42], [43]. Up-regulation of *MDM2* was also observed in response to AFB_1_ exposure in swine [19] and this may be a conserved response to AFB_1_ exposure across diverse species, including poultry. Misregulation of *MDM2* could play a role in hyperplasia and other liver remodeling processes by over-inhibiting tumor suppressors and apoptotic pathways and decreasing control of the cell cycle.

Another gene up-regulated in hepatic responses to AFB_1_ was osteopontin (*OPN*), which encodes an extracellular matrix glycoprotein produced by both immune cells and tumor cells. In mammalian liver, OPN acts as a signaling molecule and has been linked to inflammation, leukocyte infiltration, fibrosis, and carcinogenesis [44], [45]. Unlike the turkey, *OPN* expression was down-regulated in the chicken liver after AFB_1_ exposure [13]. This difference may be a result of the increased sensitivity of turkeys to AFB_1_ toxicity [3], [5]. AFB_1_ can also turn on expression of genes not found in untreated birds (i.e. the CNTL group). *MAT2A* was expressed only in the AFB and PBAFB treatment groups. In humans, two genes, *MAT1A* and *MAT2A*, encode interchangeable synthase subunits that produce *S*-adenosylmethionine, which is involved in hepatocyte growth and apoptosis [46]. In normal mammalian hepatocytes, only *MAT1A* is expressed, while development of HCC turns on *MAT2A* expression in place of *MAT1A*. *MAT1A* has been shown to be down-regulated after AFB_1_ consumption in pigs [19]. Up-regulation of *MDM2*, *OPN* and *MAT2A* in the turkey appears to participate in the proliferative phenotype in the liver after AFB_1_ exposure.

### AFB_1_ and Lipids

AFB_1_ exposure also changes lipid metabolism and causes steatosis in the liver. In turkeys and chickens, increased lipid content often causes liver pigmentation to become pale or yellowed [5], [35], [47]. This change arises from an increase in lipid-containing vacuoles in hepatocytes [5], [6], [35]. Pale livers were observed in turkeys from the AFB group [18] and significant DE was identified for multiple genes involved in lipid regulation. *LPL* and *MID1IP1* were significantly down-regulated only in the AFB group. A similar decrease in *LPL* expression was observed in the liver of chickens exposed to AFB_1_ [13]. As a lipase, LPL is involved in the breakdown of triglycerides in lipoproteins and essential to lipid metabolism and storage. High hepatic LPL activity and mRNA expression have been linked to liver steatosis in humans and mice [48], [49]. Lower plasma LPL activity and increased hepatic expression have also been correlated with higher lipid storage in livers from certain breeds of geese [50], [51]. AFB_1_ has an opposite effect on *LPL* expression, but still induces fatty change in hepatocytes. AFB_1_ impacts on LPL activity in the liver and periphery and the mechanism of lipid accumulation in the liver need to be elucidated. In mammals, MID1IP1 is a regulator of lipogenesis that turns on fatty acid synthesis through activation of acetyl-coA carboxylase [52], [53]. Down-regulation of this lipogenic protein in the turkey could be a hepatic response to the increased retention of lipids in the liver.

### Impact of Probiotics

Previous research has identified many beneficial probiotics useful as feed additives in poultry, including strains of *Lactobacillus*, *Bifidobacteria* and *Propionibacteria* [54], [55], [56]. In this challenge study, probiotic treatment significantly decreased expression of many transcripts. Reduced expression of genes involved in metabolic processes, such as *A2M*, *ENDOU*, and serine racemase (*SRR*), could limit the biosynthetic capabilities of the liver. Examining the liver transcriptome of the PBAFB group allows for evaluation of the efficacy of the PB treatment in reducing aflatoxicosis. PBAFB treatment decreased the number of transcripts with significant DE compared to AFB_1_ treatment alone and led to normal liver weights and weight gains [18]. However, the levels of hepatocyte necrosis and biliary hyperplasia were not reduced [18]. Most transcripts with significant DE in both the AFB and PBAFB groups had a higher log_2_ fold change in the PBAFB group, suggesting a synergistic effect. Additionally, approximately 70 transcripts had significant DE only in the PBAFB group, further suggesting an interaction between these treatments. Therefore, the addition of PB does modulate the effects of AFB_1_, but expression levels for many transcripts do not resemble the CNTL group. Although full mitigation of AFB_1_ toxicity was not expected, treatment with probiotics was not as protective as might be predicted. Further experiments would be needed to determine if higher concentrations or different compositions of dietary probiotics can reduce hepatic lesions and gene expression changes caused by AFB_1_.

## Conclusions

General characterization of liver transcriptome dynamics in response to toxicological challenge with AFB_1_ was achieved by RNA-seq in the turkey. Transcriptome analysis identified genes involved in responses to AFB_1_, genes that were misregulated as a result of toxicity, and genes modulated by the probiotics. These genes provide a list of targets for further investigation of AFB_1_ toxicity in the turkey liver. *MDM2*, *OPN* and other genes linked to cancer provide evidence for the apoptotic and cell cycle regulatory pathways that are likely the molecular mechanisms of inflammation, proliferation and liver damage in aflatoxicosis. Further investigation of these pathways at the cellular level would be beneficial to both basic understanding of aflatoxicosis and applications to reduce toxic processes. Regulatory and signaling genes like *OPN* could be useful for direct modulation of responses to toxicity. Genes such as *LPL*, *MAT2A*, and *MDM2* could be utilized as biomarkers for AFB_1_ exposure and aflatoxicosis in flocks and in efficacy testing for potential toxicity reduction strategies.

Investigating aflatoxicosis through transcriptome sequencing provides a powerful approach for research aiming to reduce poultry susceptibility to AFB_1_. In this context, RNA-seq would be an effective tool for future toxicity studies. By examining the transcriptomes of the spleen, kidney, muscle and other tissues, the systemic effects of AFB_1_ exposure could be further elucidated. Comparative analysis of other galliform species could also distinguish differences in AFB_1_ response, beyond the known variation in liver bioprocessing [57], [58]. Characterizing variation in transcriptome responses to AFB_1_ and to other mycotoxins and dietary contaminants could allow for the identification of protective alleles with the potential to mitigate the effects of aflatoxicosis.

## Supporting Information

Figure S1 **Average quality scores per read for RNA-seq datasets after filtering and trimming.** Quality scores were averaged and the number of reads totaled by score with FastQC [22]. Quality scores were plotted against read counts for the cumulative liver data (black), as well as each treatment dataset. The control (CNTL) (green) and aflatoxin B_1_ (AFB) (blue) samples were run on flow cell 1, while the probiotic mixture (PB) (red) and probiotic + aflatoxin B_1_ (PBAFB) (purple) were run on flow cell 2.
(TIF)

Figure S2 **Quality scores at each base position for RNA-seq datasets after filtering and trimming.** Box-plots were generated using FastQC [22]. The red line represents the median and the blue line the mean at each base. (A) Control (CNTL). (B) Aflatoxin B_1_ (AFB). (C) Probiotic mixture (PB). (D) Probiotic + aflatoxin B_1_ (PBAFB).
(TIF)

Figure S3 **Depth of coverage on predicted transcripts for each treatment group.** The number of transcripts was plotted for each level of read coverage for the control (CNTL) (green), aflatoxin B_1_ (AFB) (blue), probiotic (PB) (red) and probiotic + aflatoxin B_1_ (PBAFB) (purple) groups. A threshold of 0.1 read/million mapped was used to filter transcripts for coverage. The minimum read depth to meet this threshold varied most between treatments on flow cell 1 (long dash) and flow cell 2 (short dash) due to different library sizes.
(TIF)

Figure S4 **Histogram of** ***de novo*** **assembled transcript lengths after coverage threshold filtering.** Each bin represents the number of filtered transcripts with a length less than or equal to the bin value, but greater than the previous bin.
(TIF)

Figure S5 **Pair-wise comparisons of mean expression and log_2_ FC between treatments.** Each plot shows log_2_ fold change (FC) against mean normalized expression for predicted transcripts with non-zero expression values in both treatments generated in DESeq [27]. Transcripts with significant differential expression (DE) (q-values ≤0.05) are highlighted in red. (A). Probiotic mixture (PB) to control (CNTL). (B) Probiotic + aflatoxin B_1_ (PBAFB) to CNTL. (C) PBAFB to aflatoxin B_1_ (AFB). (D) PBAFB to PB.
(TIF)

Figure S6 **Box-plots of log_2_ FC for transcripts with significant DE in each pair-wise comparison.** Each plot shows the distribution of log_2_ fold change (FC) for transcripts with significant differential expression (DE) (q- value ≤0.05) between treatments and with non-zero normalized expression values in both treatments. Treatments with significantly different mean log_2_ FC (p-value ≤0.05) are indicated by an *. Outliers are illustrated by open circles. (A) Log_2_ FC for significant transcripts in each treatment compared to the control (CNTL). (B) Log_2_ FC for significant transcripts in the probiotic + aflatoxin B_1_ (PBAFB) group compared to the aflatoxin B_1_ (AFB) or probiotic mixture (PB) group.
(TIF)

Figure S7 **Relationship between log_2_ FC and significance level for each pair-wise comparison.** Each volcano plot shows –log_10_ p-value against log_2_ fold change (FC) for predicted transcripts expressed in both treatments. Transcripts with significant differential expression (DE) (q-value ≤0.05) are highlighted in red. (A) Aflatoxin B_1_ (AFB) to control (CNTL). (B) Probiotic mixture (PB) to CNTL. (C) Probiotic + aflatoxin B_1_ (PBAFB) to CNTL. (D) PBAFB to AFB. (E) PBAFB to PB.
(TIF)

Figure S8 **Biological process GO terms associated with significant DE transcripts in PBAFB inter-treatment comparisons.** Using BLAST2GO [32], level 2 biological process Gene Ontology (GO) terms were identified for transcripts with significant differential expression (DE) in the probiotic + aflatoxin B_1_ (PBAFB) group when compared to the aflatoxin B_1_ (AFB) or probiotic mixture (PB) group. The distribution of associated GO terms for these significant transcripts was plotted as the percent of total associations.
(TIF)

Table S1 **Results of filtering predicted liver transcripts by a coverage threshold (0.1 read/million).**
(DOCX)

Table S2 **Distribution of filtered transcripts across the turkey genome (build UMD 2.01).**
(DOCX)

Table S3 **Characterization of predicted transcripts with significant DE identified using DESeq.** Results of differential expression (DE) analysis in DESeq [27] were compiled with read counts from HTSeq [26], BLAST annotations, and Gene Ontology (GO) terms from BLAST2GO [32]. Each tab represents a pair-wise comparison between treatment groups. Raw and normalized expression values, fold change (FC), log_2_ FC, p-values, q-values (FDR-adjusted p-values), top BLAST hits for the turkey, chicken, Swiss-Prot and non-redundant (NR) databases, and GO terms are shown for each significant transcript.
(XLSX)

Checklist S1 **ARRIVE Checklist.**
(DOC)
