# Supplementary figures and images for: Response of the Hepatic Transcriptome to Aflatoxin B1 in Domestic Turkey (Meleagris gallopavo)
Source: PLoS One. 2014 Jun 30;9(6):e100930. doi: 10.1371/journal.pone.0100930 (PMC4076218; doi:10.1371/journal.pone.0100930)

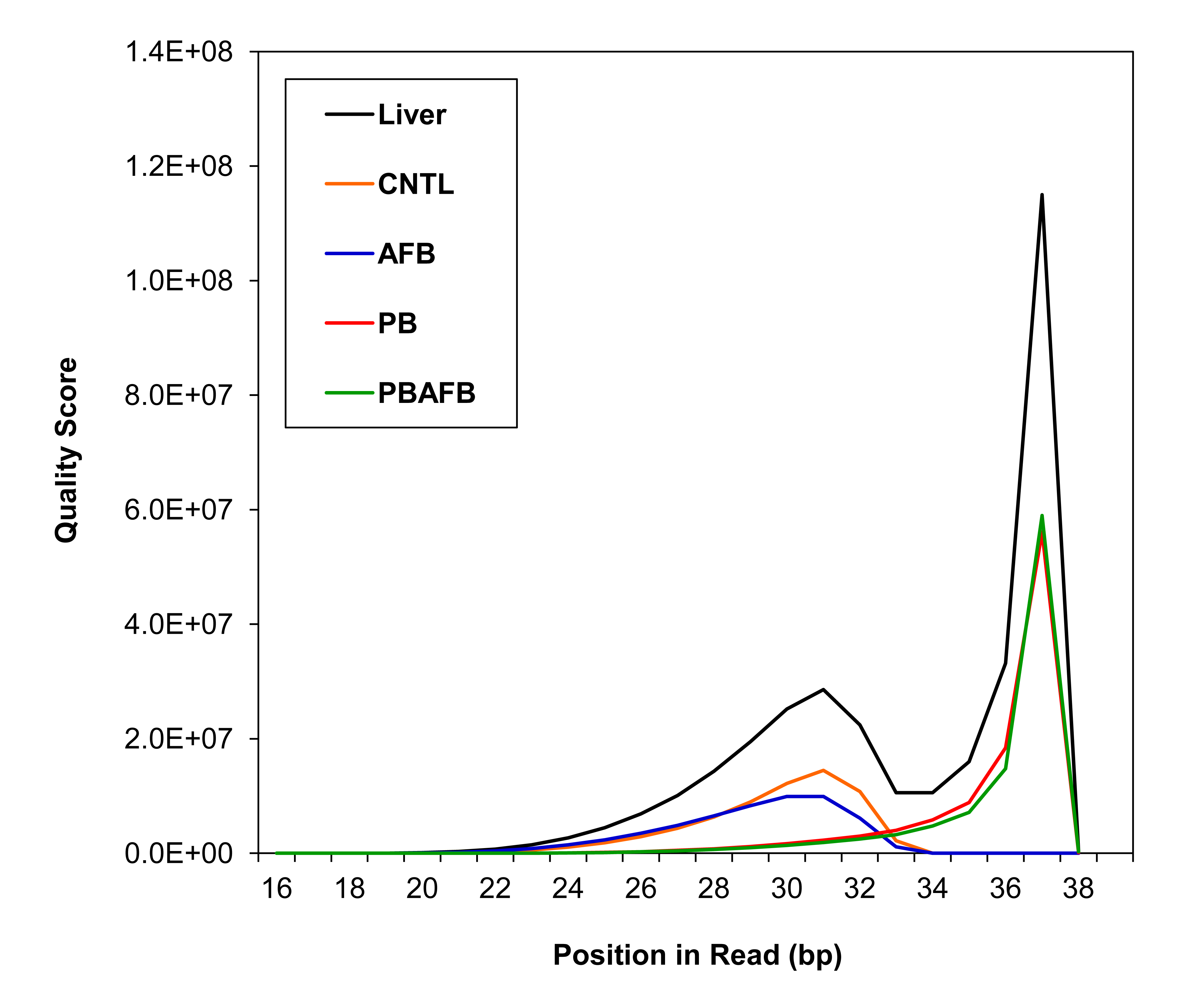

Supplement: Figure S1 — Average quality scores per read for RNA-seq datasets after filtering and trimming. Quality scores were averaged and the number of reads totaled by score with FastQC [22]. Quality scores were plotted against read counts for the cumulative liver data (black), as well as each treatment dataset. The control (CNTL) (green) and aflatoxin B1 (AFB) (blue) samples were run on flow cell 1, while the probiotic mixture (PB) (red) and probiotic + aflatoxin B1 (PBAFB) (purple) were run on flow cell 2. (TIF) [file pone.0100930.s001.tif]

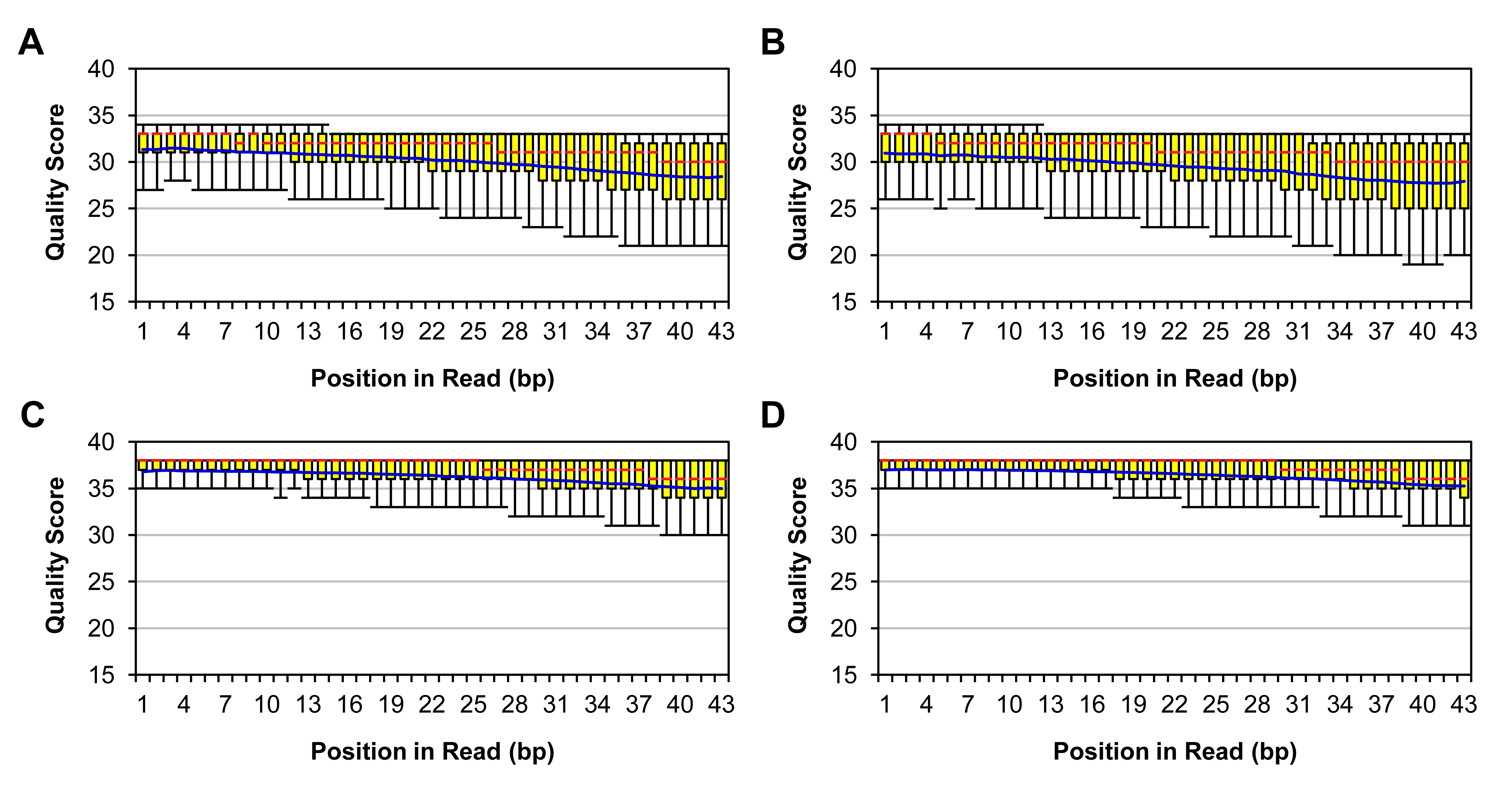

Supplement: Figure S2 — Quality scores at each base position for RNA-seq datasets after filtering and trimming. Box-plots were generated using FastQC [22]. The red line represents the median and the blue line the mean at each base. (A) Control (CNTL). (B) Aflatoxin B1 (AFB). (C) Probiotic mixture (PB). (D) Probiotic + aflatoxin B1 (PBAFB). (TIF) [file pone.0100930.s002.tif]

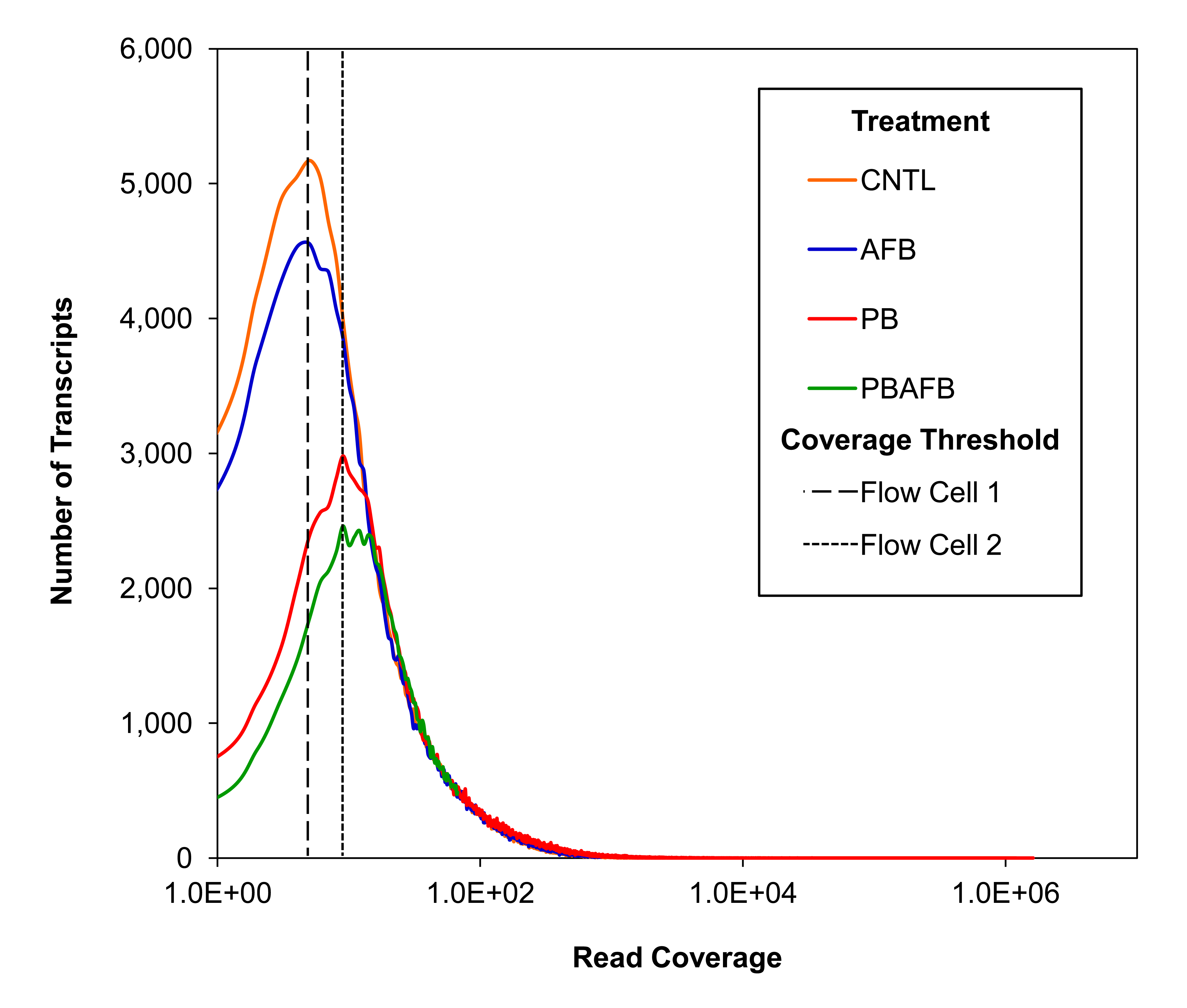

Supplement: Figure S3 — Depth of coverage on predicted transcripts for each treatment group. The number of transcripts was plotted for each level of read coverage for the control (CNTL) (green), aflatoxin B1 (AFB) (blue), probiotic (PB) (red) and probiotic + aflatoxin B1 (PBAFB) (purple) groups. A threshold of 0.1 read/million mapped was used to filter transcripts for coverage. The minimum read depth to meet this threshold varied most between treatments on flow cell 1 (long dash) and flow cell 2 (short dash) due to different library sizes. (TIF) [file pone.0100930.s003.tif]

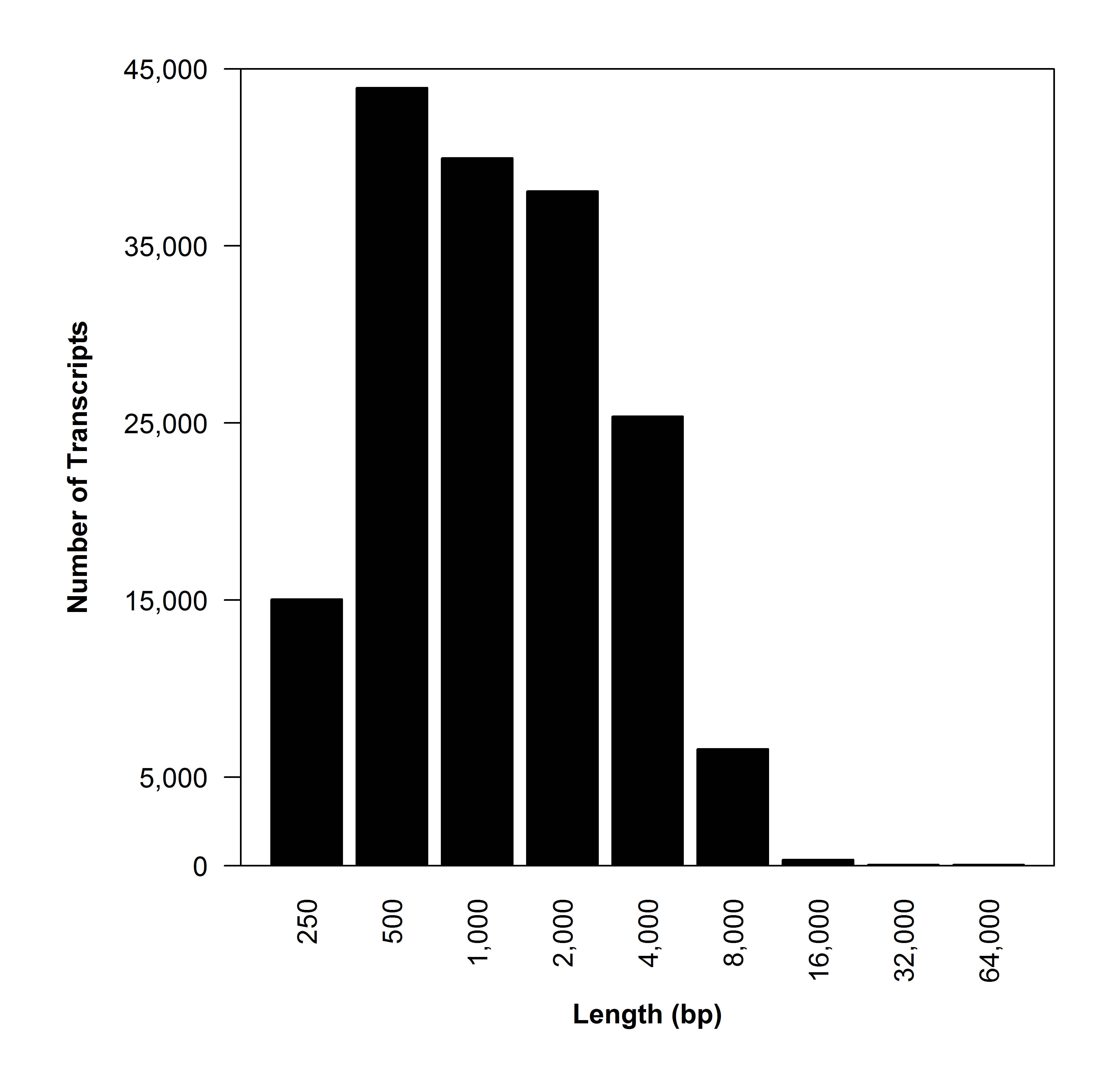

Supplement: Figure S4 — Histogram of de novo assembled transcript lengths after coverage threshold filtering. Each bin represents the number of filtered transcripts with a length less than or equal to the bin value, but greater than the previous bin. (TIF) [file pone.0100930.s004.tif]

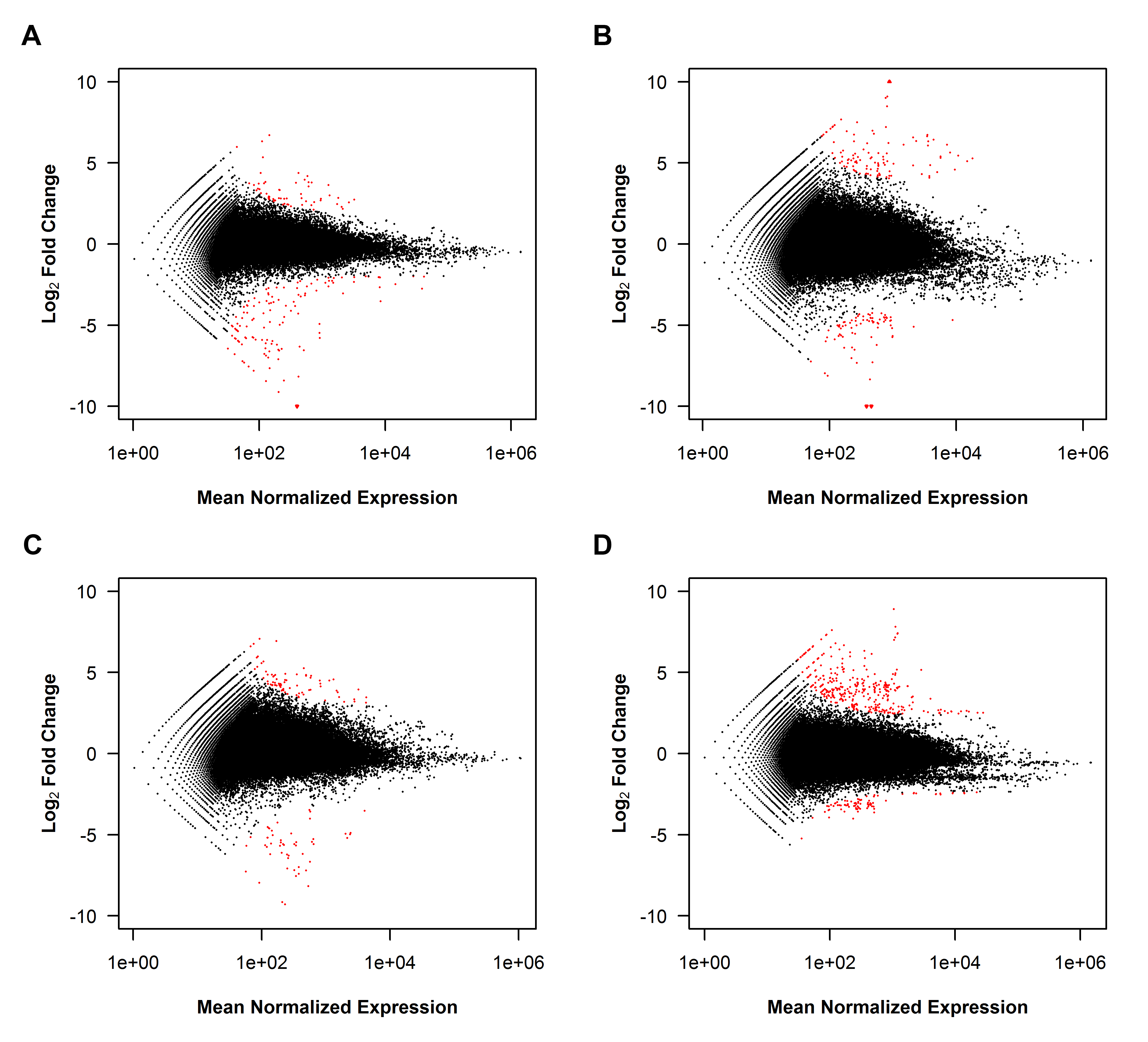

Supplement: Figure S5 — Pair-wise comparisons of mean expression and log2 FC between treatments. Each plot shows log2 fold change (FC) against mean normalized expression for predicted transcripts with non-zero expression values in both treatments generated in DESeq [27]. Transcripts with significant differential expression (DE) (q-values ≤0.05) are highlighted in red. (A). Probiotic mixture (PB) to control (CNTL). (B) Probiotic + aflatoxin B1 (PBAFB) to CNTL. (C) PBAFB to aflatoxin B1 (AFB). (D) PBAFB to PB. (TIF) [file pone.0100930.s005.tif]

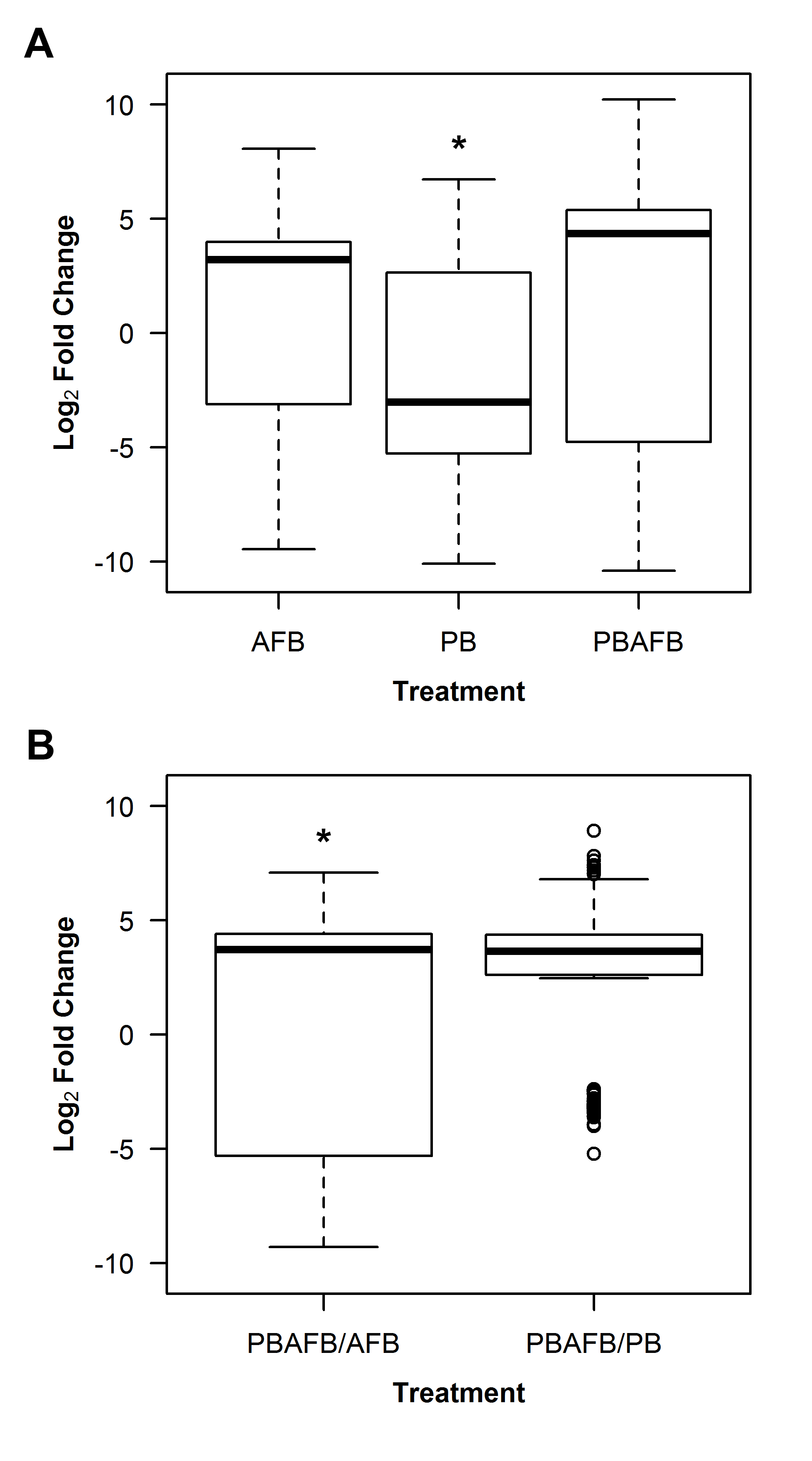

Supplement: Figure S6 — Box-plots of log2 FC for transcripts with significant DE in each pair-wise comparison. Each plot shows the distribution of log2 fold change (FC) for transcripts with significant differential expression (DE) (q- value ≤0.05) between treatments and with non-zero normalized expression values in both treatments. Treatments with significantly different mean log2 FC (p-value ≤0.05) are indicated by an *. Outliers are illustrated by open circles. (A) Log2 FC for significant transcripts in each treatment compared to the control (CNTL). (B) Log2 FC for significant transcripts in the probiotic + aflatoxin B1 (PBAFB) group compared to the aflatoxin B1 (AFB) or probiotic mixture (PB) group. (TIF) [file pone.0100930.s006.tif]

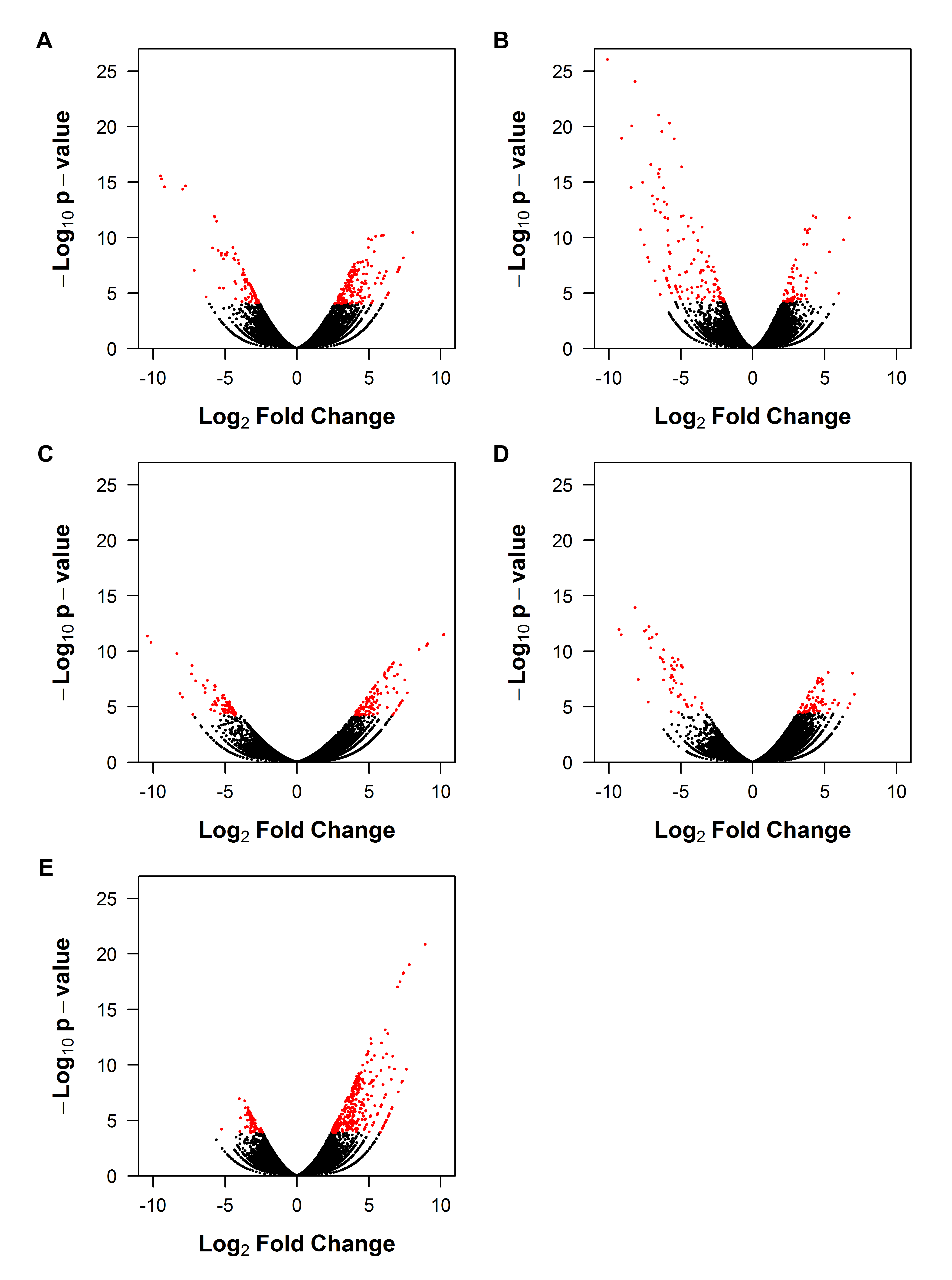

Supplement: Figure S7 — Relationship between log2 FC and significance level for each pair-wise comparison. Each volcano plot shows –log10 p-value against log2 fold change (FC) for predicted transcripts expressed in both treatments. Transcripts with significant differential expression (DE) (q-value ≤0.05) are highlighted in red. (A) Aflatoxin B1 (AFB) to control (CNTL). (B) Probiotic mixture (PB) to CNTL. (C) Probiotic + aflatoxin B1 (PBAFB) to CNTL. (D) PBAFB to AFB. (E) PBAFB to PB. (TIF) [file pone.0100930.s007.tif]

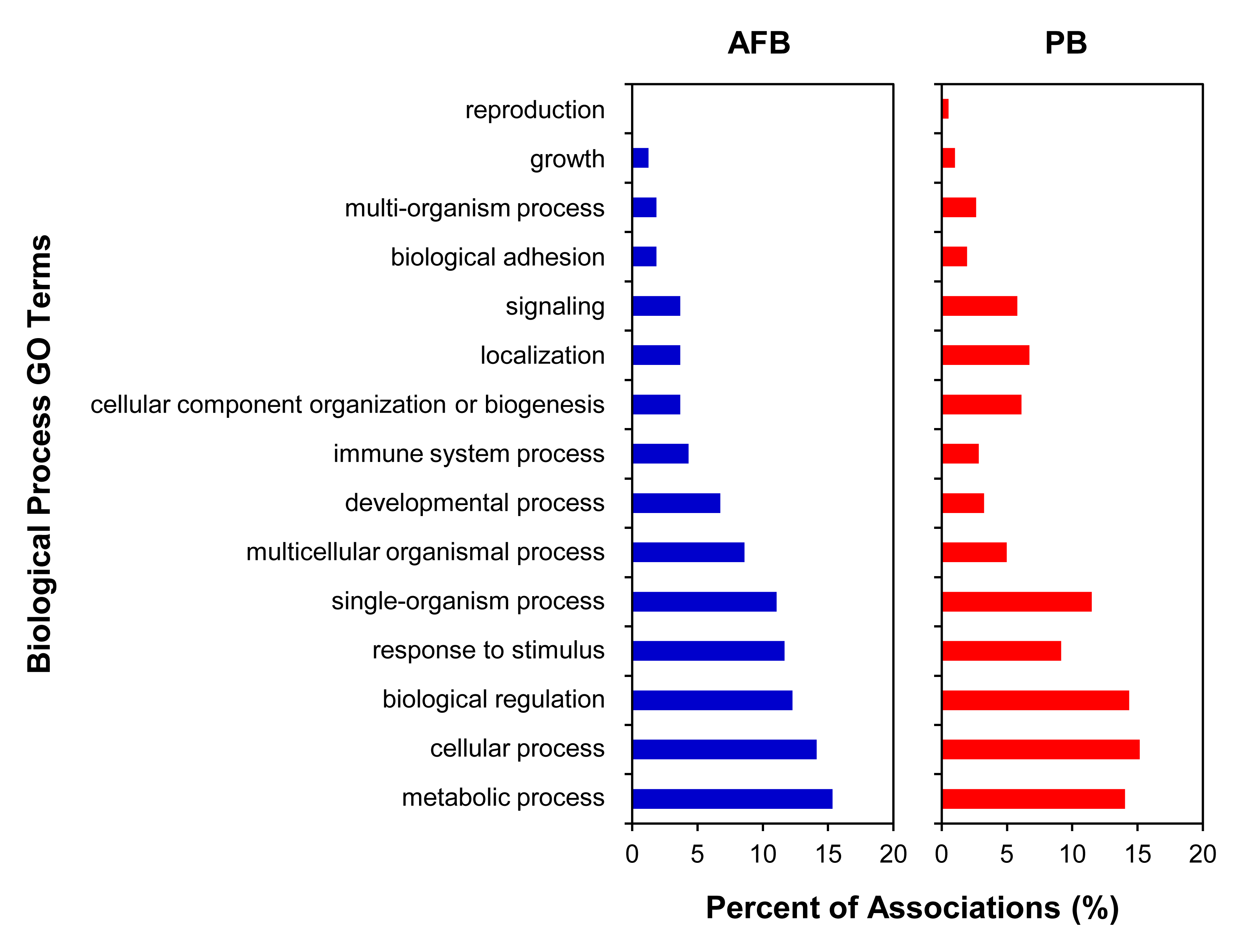

Supplement: Figure S8 — Biological process GO terms associated with significant DE transcripts in PBAFB inter-treatment comparisons. Using BLAST2GO [32], level 2 biological process Gene Ontology (GO) terms were identified for transcripts with significant differential expression (DE) in the probiotic + aflatoxin B1 (PBAFB) group when compared to the aflatoxin B1 (AFB) or probiotic mixture (PB) group. The distribution of associated GO terms for these significant transcripts was plotted as the percent of total associations. (TIF) [file pone.0100930.s008.tif]
