# Supplementary material for: Response of the Hepatic Transcriptome to Aflatoxin B1 in Domestic Turkey (Meleagris gallopavo)
Source: PLoS One. 2014 Jun 30;9(6):e100930. doi: 10.1371/journal.pone.0100930 (PMC4076218; doi:10.1371/journal.pone.0100930)
Supplement: Table S1 — Results of filtering predicted liver transcripts by a coverage threshold (0.1 read/million). (DOCX) [file pone.0100930.s009.docx]

**Table S1.** Results of filtering predicted liver transcripts by a coverage threshold (0.1 read/million).

|  | |  |  |  |  |  |
| --- | --- | --- | --- | --- | --- | --- |
|  | | **CNTL** | **AFB** | **PB** | **PBAFB** | **Total** |
| Minimum Coverage to Meet Threshold^1^ | | 6 reads | 5 reads | 10 reads | 9 reads | N/A^2^ |
| Predicted Transcripts | Total Expressed | 172,302 | 172,538 | 173,853 | 173,643 | 174,010 |
|  | Above Threshold | 149,864 (87.0%) | 157,314 (91.2%) | 154,837 (89.1%) | 161,751 (93.2%) | 169,387 (97.3%) |
|  | Below Threshold | 22,438 (13.0%) | 15,224 (8.8%) | 19,016 (10.9%) | 11,892 (6.8%) | 4,623 (2.7%) |
| Mapped Reads^3^ | Total Mapped | 63,137,343 | 52,619,894 | 98,770,095 | 92,577,894 | 307,105,226 |
|  | Above Threshold | 63,065,160 | 52,578,880 | 98,659,516 | 92,512,284 | 306,815,840 |
|  | Below Threshold | 72,183 | 41,014 | 110,579 | 65,610 | 289,386 |

^1^ Minimum read depth was calculated separately for the control (CNTL), aflatoxin B_1_ (AFB), probiotic mixture (PB), and probiotic + aflatoxin B_1_ (PBAFB) groups. The number of reads mapped to a transcript must be ≥ to these values to meet the threshold.

^2^ Not applicable (N/A).

^3^ In all treatments, 99.9% of mappable reads aligned to transcripts that met the coverage threshold and 0.1% to transcripts below.
