# Supplementary material for: Response of the Hepatic Transcriptome to Aflatoxin B1 in Domestic Turkey (Meleagris gallopavo)
Source: PLoS One. 2014 Jun 30;9(6):e100930. doi: 10.1371/journal.pone.0100930 (PMC4076218; doi:10.1371/journal.pone.0100930)
Supplement: Table S2 — Distribution of filtered transcripts across the turkey genome (build UMD 2.01). (DOCX) [file pone.0100930.s010.docx]

**Table S2.** Distribution of filtered transcripts across the turkey genome (build UMD 2.01).

|  |  |  |  |
| --- | --- | --- | --- |
| **Chromosome** | **Number of Transcripts Mapped** | **Chromosome Size (bp) (Excluding Gaps)** | **Transcripts/Mb** |
| 1 | 28,638 | 181,826,552 | 157.5 |
| 2 | 14,859 | 106,718,223 | 139.2 |
| 3 | 10,339 | 91,132,767 | 113.4 |
| 4 | 11,005 | 68,844,569 | 159.9 |
| 5 | 11,665 | 56,965,239 | 204.8 |
| 6 | 6,541 | 48,705,183 | 134.3 |
| 7 | 7,253 | 35,338,084 | 205.2 |
| 8 | 7,222 | 35,279,744 | 204.7 |
| 9 | 3,408 | 18,014,631 | 189.2 |
| 10 | 7,052 | 28,668,829 | 246.0 |
| 11 | 5,371 | 22,659,912 | 237.0 |
| 12 | 4,306 | 18,944,919 | 227.3 |
| 13 | 3,895 | 18,696,996 | 208.3 |
| 14 | 3,927 | 19,181,786 | 204.7 |
| 15 | 4,191 | 16,791,072 | 249.6 |
| 16 | 4,868 | 14,411,805 | 337.8 |
| 17 | 4,255 | 12,015,459 | 354.1 |
| 18 | 820 | 139,801 | 5,865.5 |
| 19 | 3,304 | 9,478,246 | 348.6 |
| 20 | 3,716 | 9,943,105 | 373.7 |
| 21 | 3,558 | 9,405,728 | 378.3 |
| 22 | 3,236 | 13,252,797 | 244.2 |
| 23 | 2,688 | 6,420,024 | 418.7 |
| 24 | 992 | 3,616,665 | 274.3 |
| 25 | 2,082 | 4,963,017 | 419.5 |
| 26 | 1,591 | 5,925,429 | 268.5 |
| 27 | 567 | 687,724 | 824.5 |
| 28 | 2,583 | 4,244,239 | 608.6 |
| 29 | 2,040 | 3,649,262 | 559.0 |
| 30 | 2,264 | 3,524,564 | 642.3 |
| W | 32 | 108,225 | 295.7 |
| Z | 9,515 | 47,725,835 | 199.4 |
| Total | 151,492 | 917,280,431 | 165.2 |
| Multi-Mapping | 21,281 | N/A^1^ | N/A |
| Unmapped | 17,895 | N/A | N/A |

^1^ Not applicable (N/A).
